# Establishing research priorities relating to the long-term impact of TIA and minor stroke through stakeholder-centred consensus

**DOI:** 10.1186/s40900-018-0089-z

**Published:** 2018-01-25

**Authors:** Grace M. Turner, Ruth Backman, Christel McMullan, Jonathan Mathers, Tom Marshall, Melanie Calvert

**Affiliations:** 1 Institute of Applied Health Research, University of Birmingham, Edgbaston, Birmingham, B15 2TT England; 2 Centre for Patient Reported Outcomes Research, University of Birmingham, Edgbaston, Birmingham, B15 2TT England

**Keywords:** Transient ischemic attack, Minor stroke, Research priorities, Stakeholder engagement, Long term effects, United Kingdom

## Abstract

**Plain English summary:**

**What is the problem and why is this important?**

Mini-strokes are similar to full strokes, but symptoms last less than 24 h. Many people (up to 70%) have long-term problems after a mini-stroke, such as anxiety; depression; problems with brain functioning (like memory loss); and fatigue (feeling tired). However, the current healthcare pathway only focuses on preventing another stroke and care for other long-term problems is not routinely given. Without proper treatment, people with long-term problems after a mini-stroke could have worse quality of life and may find it difficult to return to work and their social activities.

**What is the aim of the research?**

We wanted to understand the research priorities of patients, health care professionals and key stakeholders relating to the long-term impact of mini-stroke.

**How did we address the problem?**

We invited patients, clinicians, researchers and other stakeholders to attend a meeting. At the meeting people discussed the issues relating to the long-term impact of mini-stroke and came to an agreement on their research priorities. There were three stages: (1) people wrote down their individual research suggestions; (2) in smaller groups people came to an agreement on what their top research questions were; and (3) the whole group agreed final research priorities.

**What did we find?**

Eleven people attended who were representatives for patients, GPs, stroke consultants, stroke nurses, psychologists, the Stroke Association (charity) and stroke researchers, The group agreed on eleven research questions which they felt were the most important to improve health and well-being for people who have had a mini-stroke.

The eleven research questions encompass a range of categories, including: understanding the existing care patients receive (according to diagnosis and geographical location); exploring what optimal care post-TIA/minor stroke should comprise (identifying and treating impairments, information giving and support groups) and how that care should be delivered (clinical setting and follow-up pathway); impact on family members; and education/training for health care professionals.

**Abstract:**

**Background** Clinical management after transient ischaemic attack (TIA) and minor stroke focuses on stroke prevention. However, evidence demonstrates that many patients experience ongoing residual impairments. Residual impairments post-TIA and minor stroke may affect patients’ quality of life and return to work or social activities. Research priorities of patients, health care professionals and key stakeholders relating to the long-term impact of TIA and minor stroke are unknown.

**Methods** Our objective was to establish the top shared research priorities relating to the long-term impact of TIA and minor stroke through stakeholder-centred consensus. A one-day priority setting consensus meeting took place with representatives from different stakeholder groups in October 2016 (Birmingham, UK). Nominal group technique was used to establish research priorities. This involved three stages: (i) gathering research priorities from individual stakeholders; (ii) interim prioritisation in three subgroups; and (iii) final priority setting.

**Results** The priority setting consensus meeting was attended by 11 stakeholders. The individual stakeholders identified 34 different research priorities. During the interim prioritisation exercise, the three subgroups generated 24 unique research priorities which were discussed as a whole group. Following the final consensus discussion, 11 shared research priorities were unanimously agreed.

The 11 research questions encompass a range of categories, including: understanding the existing care patients receive (according to diagnosis and geographical location); exploring what optimal care post-TIA/minor stroke should comprise (identifying and treating impairments, information giving and support groups) and how that care should be delivered (clinical setting and follow-up pathway); impact on family members; and education/training for health care professionals.

**Conclusions** Eleven different research priorities were established through stakeholder-centred consensus. These research questions could usefully inform the research agenda and policy decisions for TIA and minor stroke. Inclusion of stakeholders in setting research priorities is important to increase the relevance of research and reduce research waste.

## Background

Transient ischaemic attack (TIA) occurs when there is a transient disruption of blood supply to the brain, caused by a temporary blood clot [1]. Symptoms are similar to stroke but short lasting, usually < 1 h [1]. In contrast, minor stroke refers to an episode where obstruction of the blood supply to the brain causes tissue death in the brain, but symptoms are non-disabling and people experience short-term functional recovery [2]. Both TIA and minor stroke patients functionally recover well and arguably these diagnoses are a continuum of each other.

TIAs and minor strokes are common. Over 510,000 people in the United Kingdom (UK) have experienced a TIA or minor stroke [3] and the incidence is rising [4]. Clinical management post-TIA and minor stroke focuses on stroke prevention; however, evidence demonstrates that many patients experience ongoing residual impairments [5–7]. A retrospective analysis of UK primary care records found that TIA patients had increased risk of general practitioners (GPs) consultations for fatigue, anxiety, depression and cognitive impairment, compared to matched controls [7]. Furthermore, two recent systematic reviews have found relatively high prevalence of these impairments post-TIA and minor stroke [5, 8].

Despite good functional recovery, ‘hidden’ impairments post-TIA and minor stroke may affect patients’ quality of life (QoL) [9–14]. Evidence also suggests that TIA and minor stroke are associated with non-return to work, [9, 10, 15–17] decreased life satisfaction and reduced participation in meaningful activities, such as basic self-care, recreational activities and driving [13, 15, 16, 18]. Furthermore, reduced QoL post-TIA and minor stroke has been found to be associated with stroke recurrence [19].

In 2014 UKs leading stroke charity, the Stroke Association, called for “further research into the long-term effects of TIA” [6]. However, few studies that have evaluated interventions to treat/ manage impairments or improve QoL post-TIA/minor stroke. Our review of the literate identified nine relevant studies which comprised interventions of: education and aerobic exercise (*n* = 6); [20–25] education (*n* = 1); [26] exercise (*n* = 1); [27] and multimodal (telephone, internet and paper) support (*n* = 1) [28]. Over half of these interventions [20, 23–25, 28] did not primarily aim to improve residual impairments or QoL; primary outcomes were vascular risk factors and unplanned use of the healthcare system. Furthermore, only one study (a protocol) is based in the UK [26] (Canada *n* = 3, Netherlands *n* = 1, [27] Norway *n* = 1, [21] Switzerland *n* = 1, [22] New Zealand *n* = 1, [20] Ireland *n* = 1 [25]). Therefore, there is a need to address the long-term effects of TIA/ minor stroke in a UK setting.

Research priorities of patients, health care professionals and key stakeholders are unknown. Involvement of these stakeholders in shaping the research agenda is important to ensure that research is relevant and applicable to the users of research and, therefore, more likely to be implemented in practice [29]. It is also important to promote partnership with these stakeholders and encourage engagement with research [29]. Furthermore, Chalmers et al. [30] suggests that there is often a mismatch between the priorities of researchers and the priorities of the end users of researchers. They recommend that patients and health care professionals are consulted during the setting of research agendas to increase value and reduce research waste [30]. Our objective was to establish the top shared research priorities relating to the long-term impact of TIA and minor stroke through stakeholder-centred consensus.

## Methods

A one-day priority setting consensus meeting took place with representatives from different stakeholder groups in October 2016 (Birmingham, UK). The aim of the event was to establish the top shared research priorities relating to the long-term impact of TIA and minor stroke. A one-day event has the advantage of efficient us of time and resources and reduced burden on attendees.

A number of strategies were used to recruit stakeholders, including: adverts on patient websites (including People in Research, Different Strokes, University of the Third Age); presentations at community events (such as the Birmingham and Black Country Stroke Research Awareness Day) email invites sent to relevant stakeholders identified through internet searches and known contacts; and snowballing. Stakeholders included people with a lived experience of TIA or minor stroke, healthcare professionals, stroke charities and stroke researchers. The aim was to establish the top, shared research priorities relating to the long-term impact of TIA and minor stroke. ‘Long-term impact’ was defined as post-diagnosis of TIA or minor stroke with no restrictions on length of time after the event.

Nominal group technique was used to establish research priorities. This involved three stages: (i) gathering research priorities from individual stakeholders; (ii) interim prioritisation; and (iii) final priority setting.

### Gathering research priorities

Prior to the meeting, individual stakeholders were invited to list unanswered questions about life after TIA or minor stroke using free text questionnaires. This generated a long list of research priorities, only duplicated research priorities were removed.

### Interim prioritisation

Interim prioritisation of research questions was conducted in in three subgroups comprising 3-4 stakeholders. The groups discussed their individual research priorities and generated a list of shared group priorities which they then ranked in order of priority. Three researchers (GT, CM and RB) facilitated the subgroup discussion.

### Final priority setting

Following interim prioritisation, the sub-groups presented their ranked research questions and there was a while group discussion to compare and debate outcomes of the three groups. The lead researcher (GT) facilitated the discussion. As a whole group, a final consensus on of the shared priority areas for research was agreed.

### Data collection and ethical approval

Discussions were audio-recorded using an encrypted digital recorder and nominated scribes documented the research priorities. Ethical consent was gained from the University of Birmingham’s Science, Technology, Engineering and Mathematics Ethical Review Committee (reference: ERN_16-1057). Written consent was obtained from all stakeholders prior to the meeting.

## Results

The priority setting consensus meeting was attended by 11 stakeholders: three TIA/minor stroke patients; three stroke nurses; one stroke consultant; one general practitioner (GP); one psychologist; one representative from the Stroke Association (charity); and one stroke researcher (Table 1).

**Table 1:** Stakeholder characteristics (*n* = 11)

		Number
Age	< 25	0
	25-34	1
	35-44	3
	45-54	2
	55-64	3
	65-74	1
	75-84	1
	≥84	0
Sex	Male	6
Stakeholder category	Healthcare professional	5
	Experienced a TIA	3
	Researcher	1
	Psychologist	1
	Stroke Association representative	1

The individual stakeholders identified 34 different research priorities. These research priorities were discuss in three subgroups which comprised a mix of representatives from different stakeholder groups (Group 1: patient representative, stroke consultant, psychologist; Group 2: patient representative, researcher and two stroke nurses; Group 3: patient representative, stroke nurse, GP and stroke association representative). During the interim prioritisation exercise, the three subgroups generated 24 unique research priorities which were discussed as a whole group. Following the final consensus discussion, 11 shared research priorities were unanimously agreed (Table 2).

**Table 2:** Top research priorities relating to the long term impact of TIA and minor stroke: consensus from TIA/minor stroke patients and key stakeholders

Top 11 research priorities relating to the long term impact of TIA and minor stroke
• What is the most effective follow-up pathway for TIA/ minor stroke patients? • What is the best way to identify which TIA/ minor stroke patients will experience ongoing impairments? • What are the best ways to train or educate healthcare professionals to recognise and understand impairments post-TIA/minor stroke? • What are the best ways to manage and treat psychological impairment, cognitive impairment and fatigue after TIA/minor stroke? • Would support groups improve outcomes for people after TIA/minor stroke? • What is the best setting to support patients with impairments after TIA/minor stroke (such as TIA clinic, general practice, community setting)? • What advice should healthcare professions give to TIA/minor stroke patients on return to work and activities? • What information do people want to receive after they have experienced a TIA/minor stroke? • What impact does having a TIA/minor stroke have on family members, including children? • What impact does a diagnosis of ‘TIA’ vs ‘minor stroke’ have on patients and their follow-up care? • How do services which treat or manage impairments post-TIA/minor stroke differ in different regions of England?

The majority of research priorities (6/11) related to the management of residual impairments, including: effective follow-up pathways; identifying and treating impairments; the role of support groups; and educating healthcare professionals. Similarly, two priorities were associated with provision of information and advice to patients.

There were key discussions about lack of follow-up care for TIA and minor stroke patients and inconsistencies in diagnosis and treatment. There was a clear demand for a standardised follow-up pathway; however, lack of resources and organisational constraints were perceived as barriers. Lack of knowledge and guidance on what advice healthcare professionals should provide to patients about return to work and usual activities and the best strategies to identify and manage patients with impairments were identified as barriers to optimal care and, therefore, important research priorities.

## Discussion

Stakeholders were consulted to identify and prioritise unanswered research questions relating to the long-term impact of TIA and minor stroke. Eleven different research priorities were established through stakeholder-centred consensus. These research questions should be used to inform the research agenda and policy decisions for TIA and minor stroke.

The 11 research questions encompass a range of categories, including: understanding the existing care patients receive (according to diagnosis and geographical location); exploring what optimal care post-TIA/minor stroke should comprise (identifying and treating impairments, information giving and support groups) and how that care should be delivered (clinical setting and follow-up pathway); impact on family members; and education/training for health care professionals.

The top agreed research questions provide valuable insight into the priorities of key stakeholders to improve healthcare and quality of life after TIA and minor stroke. Importantly, the priority setting process highlighted that current care is inadequate and there are inconsistencies in the care patients receive and access to care even at a local level.

Researchers should use these research priorities to guide future research proposals which should be co-designed with patients and stakeholders. Furthermore, research priorities should be used by funders to help inform funding decisions. The research questions have implications for policy makers, particularly regarding implementing interventions and follow-up pathways. Therefore, it is essential for researchers to engage with and disseminate to policy and decision makers at local and national levels. In addition to addressing the top research priority questions, future research should also consider potential barriers for implementing change to healthcare post-TIA/minor stroke, particularly capacity and resource constraints.

The main strength of this research priority setting process is that there was good representation from different stakeholder groups, the majority which were patients or healthcare professionals who are the end users of research. Through allowing stakeholders to list and rank priorities individually, views of all stakeholders were represented with limited social acceptability bias. The three interim subgroups each included a patient representative and each subgroup was facilitated by a researcher to ensure that everyone had an equal voice. However, an important limitation is the relatively small sample size of stakeholders, which was restricted due to time and resource constraints for recruitment. Another limitation is that the majority of the stakeholders (9/11) were from the West Midlands; therefore, research priorities may not be generalizable to other regions. A larger, regional priority setting exercise, such as the James Lind Alliance priority setting partnership may generate more generalizable findings. However, the main advantage of a one-day priority setting consensus meeting is time and cost efficiency. The James Lind Alliance approach on average costs >£40,000 and takes between 12 to 18 months, after which research priorities may be outdated. For researchers to realistically be able to identify and act on shared research priorities, it is important to have a robust but time and resource efficient methodology.

From a research team perspective, the priority setting consensus meeting was beneficial, not only to guide the teams future research but also to develop collaborations and networks with different stakeholder groups. We recommend that other research groups adopt a similar approach to identifying research questions for other research areas. The main challenge with organising this event was recruiting stakeholders; we found that, although the event was planned with plenty of notice, work commitments and other unforeseen circumstances meant that a large number of people were unable to attend at the last minute. Therefore, we recommend for other people planning such an event to anticipate large dropout numbers (up to 50%) and over-recruit accordingly. We found that the small group discussions were essential to ensure each participant had an opportunity to discuss their priorities and the use of facilitators worked well to ensure everyone had an equal voice.

## Conclusions

Eleven different research priorities were generated through stakeholder-centred consensus. The 11 research questions encompass a range of categories, including: understanding the existing care patients receive; exploring what optimal care post-TIA/minor stroke should comprise and how that care should be delivered; impact on family members; and education/training for health care professionals. These research questions could usefully inform the research agenda for TIA/minor stroke. Inclusion of stakeholders in setting research priorities is important to increase the relevance of research and reduce research waste.

## Abbreviations

GPs: General practitioners
QoL: Quality of life
TIA: Transient ischaemic attack
UK: United Kingdom
